# Wnt/β-catenin signaling in heart regeneration

**DOI:** 10.1186/s13619-015-0017-8

**Published:** 2015-07-08

**Authors:** Gunes Ozhan, Gilbert Weidinger

**Affiliations:** 1 Izmir Biomedicine and Genome Center (iBG-izmir), Dokuz Eylul University, Inciralti-Balcova, 35340 Izmir, Turkey; 2 Department of Medical Biology and Genetics, Dokuz Eylul University Medical School, Inciralti-Balcova, 35340 Izmir, Turkey; 3 Institute for Biochemistry and Molecular Biology, Ulm University, Albert-Einstein-Allee 11, 89081 Ulm, Germany

**Keywords:** Wnt, Beta-catenin, Cardiomyocyte, sFrp, Regeneration, Heart, Fibrosis, Hypertrophy, Zebrafish

## Abstract

The ability to repair damaged or lost tissues varies significantly among vertebrates. The regenerative ability of the heart is clinically very relevant, because adult teleost fish and amphibians can regenerate heart tissue, but we mammals cannot. Interestingly, heart regeneration is possible in neonatal mice, but this ability is lost within 7 days after birth. In zebrafish and neonatal mice, lost cardiomyocytes are regenerated via proliferation of spared, differentiated cardiomyocytes. While some cardiomyocyte turnover occurs in adult mammals, the cardiomyocyte production rate is too low in response to injury to regenerate the heart. Instead, mammalian hearts respond to injury by remodeling of spared tissue, which includes cardiomyocyte hypertrophy. Wnt/β-catenin signaling plays important roles during vertebrate heart development, and it is re-activated in response to cardiac injury. In this review, we discuss the known functions of this signaling pathway in injured hearts, its involvement in cardiac fibrosis and hypertrophy, and potential therapeutic approaches that might promote cardiac repair after injury by modifying Wnt/β-catenin signaling. Regulation of cardiac remodeling by this signaling pathway appears to vary depending on the injury model and the exact stages that have been studied. Thus, conflicting data have been published regarding a potential role of Wnt/β-catenin pathway in promotion of fibrosis and cardiomyocyte hypertrophy. In addition, the Wnt inhibitory secreted Frizzled-related proteins (sFrps) appear to have Wnt-dependent and Wnt-independent roles in the injured heart. Thus, while the exact functions of Wnt/β-catenin pathway activity in response to injury still need to be elucidated in the non-regenerating mammalian heart, but also in regenerating lower vertebrates, manipulation of the pathway is essential for creation of therapeutically useful cardiomyocytes from stem cells in culture. Hopefully, a detailed understanding of the *in vivo* role of Wnt/β-catenin signaling in injured mammalian and non-mammalian hearts will also contribute to the success of current efforts towards developing regenerative therapies.

## Introduction

All organisms have evolved means of repairing tissue loss after injury or disease. In most species, healing of epidermal wounds and other epithelia is an efficient repair process, whereas the ability to recuperate the damage in other tissues varies widely. Mammals (including humans) can repair injury of skeletal muscle, regenerate large parts of the liver, and repair damage to the epithelia of the kidney and the lung but have limited regenerative capacity in other organs [1]. In contrast, other vertebrates, such as urodele amphibia (salamanders and newts) and certain teleost fish species, can completely regenerate lost limbs and tails and repair damage to the lens, the retina, and the central nervous system [2–5]. Importantly, zebrafish and newts can also replace lost heart tissue in adults. A thorough understanding of the cellular and molecular mechanisms of regeneration in urodele amphibia and fish is therefore very likely to be informative for the development of regenerative therapies in humans.

One of the pivotal signaling pathways regulating the regenerative process in many systems is the Wnt/β-catenin signaling pathway. In addition, Wnt/β-catenin signaling is activated in response to cardiac injury in adult mammals and plays important roles in hypertrophy and cardiac remodeling [6]. Here, we review the cellular mechanisms underlying heart regeneration in lower vertebrates and the known functions of Wnt/β-catenin signaling in the cardiac injury response both in mammals and in non-mammalian vertebrates.

## Mammalian heart injury responses

The adult mammalian heart has a very limited capacity to repair loss of cardiomyocytes (CMs) after infarction or cardiac overload disorders [7]. In contrast, adult zebrafish can regenerate the heart in response to several injury paradigms, including surgical removal of myocardial tissue, cryoinjury, and genetic ablation of cardiomyocytes [3, 8–12]. Intriguingly, neonatal mice can regenerate the myocardium after partial surgical resection as well, but this ability is lost by 7 days after birth [13]. During both zebrafish and neonatal mouse heart regeneration, differentiated CMs re-enter the cell cycle to proliferate and genetic lineage tracing experiments indicate that the majority of the newly forming myocardium is derived from pre-existing CMs [13–15]. Thus, zebrafish and neonatal mice regenerate lost myocardium not by differentiation of CMs from progenitor cells but by activating the re-entry of differentiated CMs into the cell cycle. In contrast, adult mammalian CMs have long been thought to be terminally differentiated and thus post-mitotic [16–18] and much of the growth of the postnatal mammalian heart occurs via enlargement of pre-existing CMs, *i.e.*, hypertrophy [18, 19]. While some studies in adult mouse and human myocardial cells have suggested that certain CMs are not terminally differentiated and can reinitiate the cell cycle under physiological or pathological conditions [20–22], others have found little evidence for CM proliferation in the adult heart [23–25]. Indeed, using transgenic mice that facilitated unambiguous identification of CM nuclei, Soonpaa and Field found that only 1 out of 180.000 adult mouse ventricular CMs incorporate [^3^H] thymidine, and this increased to only 3 in 36.000 nuclei (0.0083 %) in the injured heart [23–25]. The consensus conclusion from these and other studies is that DNA synthesis is very rare in differentiated adult rodent CMs even after injury, and CM hypertrophy after injury appears to largely occur without increase in DNA content in the adult heart [24, 26].

Nevertheless, there is good evidence that some new CMs do form during adult mammalian life, including humans. A pulse of atmospheric carbon-14 (^14^C) was generated by nuclear bomb tests during the Cold War and rapidly declined after atmospheric tests were banned. Since ^14^C makes its way through the food chain into human cells, the ^14^C levels found in DNA of human CMs corresponds to the atmospheric levels at the time when these cells were born. Bergmann and colleagues have measured the ^14^C concentrations in DNA of human myocardial cells [27]. They have found that in subjects born up to 22 years before the onset of bomb tests, ^14^C concentrations were elevated compared to the levels before the tests, indicating that myocardial cells contained DNA synthesized years after birth. Thus, human CMs are capable of renewal during adulthood [27]. CM renewal, however, is very slow as estimated from this study; 1 % of the CMs are renewed per year at the age of 25 and only 0.45 % at the age of 75. Thus, approximately 45 % of all CMs are exchanged during a human lifetime while 55 % remain from neonatal stages [27].

While it is not possible to identify the cellular source of newly forming CMs in the adult human heart, several studies in mice have addressed whether CM renewal during homeostasis and the low-rate CM replacement after cardiac injury are due to proliferation of existing CMs or due to CM differentiation from progenitor cells [26, 28–33]. Unfortunately, the conclusions drawn by several studies based on genetic lineage tracing of CMs or cardiac progenitor cells differ considerably. While most reports using different means to track the fate of differentiated CMs conclude that renewal of CMs during homeostasis is due to proliferation of existing CMs [26], one study concluded that progenitor cells contribute to CM formation after cardiac injury, since the progeny of differentiated CMs get diluted with other cells in injured hearts [28]. In contrast, other studies using lineage tracing of differentiated CMs and multi-isotope imaging mass spectrometry concluded that the limited CM replacement after injury is due to CM proliferation [29, 32]. However, c-kit-positive cardiac progenitor cells have been reported to contribute significantly to CM production after cardiac injury as well [30]. In contrast, another study found that c-kit-positive cells form negligible numbers of CMs both during homeostasis and after injury [33]. The dissimilar results obtained by these studies are a reminder of the fact that albeit genetic lineage tracing tools are the most powerful way to address questions of cellular lineage, each tool needs to be very critically evaluated. In particular, it is possible that some Cre lines are not specifically expressed in a particular cell type; Cre expression could cause toxicity, and the fact that most tools fail to label all cells of a particular cell population could result in failure to appreciate that the labeled population is actually heterogeneous. Yet, while further studies are needed to clarify how CMs are formed in the adult mammalian heart, the consensus is that the rate of CM formation is too low to result in significant myocardial regeneration after heart injury [26].

A possible reason for the failure of adult mammalian CMs to sufficiently proliferate in response to cardiac insult might be their increased DNA content. Most adult mammalian CMs contain more than two sets of chromosomes. Starting at 4 days post birth, rodent CMs grow and become binucleated with each nucleus remaining diploid [18]. In contrast, most human CMs maintain a single nucleus, which however increases its DNA content to tetraploidy or even higher ploidy [34–36]. Binucleation, as seen in rodents, likely represents an impediment to successful mitosis, although some indirect evidence for the proliferation of binucleated CMs has been reported as well [37]. Polyploidy of mononucleated cells might activate cell cycle arrest [38], yet many animal species exist as polyploids [39]; thus, non-diploid DNA content should not represent a principal impediment for cellular proliferation, although it possibly does in CMs. Intriguingly, experimental manipulation of several cell cycle proteins as well as activation of a variety of signaling pathways including Notch, neuregulin1/ErbB4, Fgf-1, Pi3K, and Akt signaling can stimulate cell cycle re-entry in differentiated mature mammalian CMs [40–45]. Yet, not all CMs that are driven by these regimes into DNA synthesis undergo cytokinesis, and at least for the case of neuregulin, it is clear that only mononucleated mouse CMs, which represent less than 10 % of adult CMs, could be stimulated. Nevertheless, constitutive activation of cyclin A2 and activation of neuregulin1/ErbB4, PI3K, and Akt have also been shown to promote cardiac repair and heart function upon myocardial infarction [41, 43, 46]. Thus, while adult mammalian hearts fail to activate a regenerative program of CM proliferation in response to myocardial injury, adult CMs appear to have no intrinsic block preventing their proliferation. This raises the possibility that therapeutic alteration of the signaling environment of the injured heart could activate compensatory proliferation of CMs.

## The Wnt/β-catenin signaling pathway

**Fig. 1:**
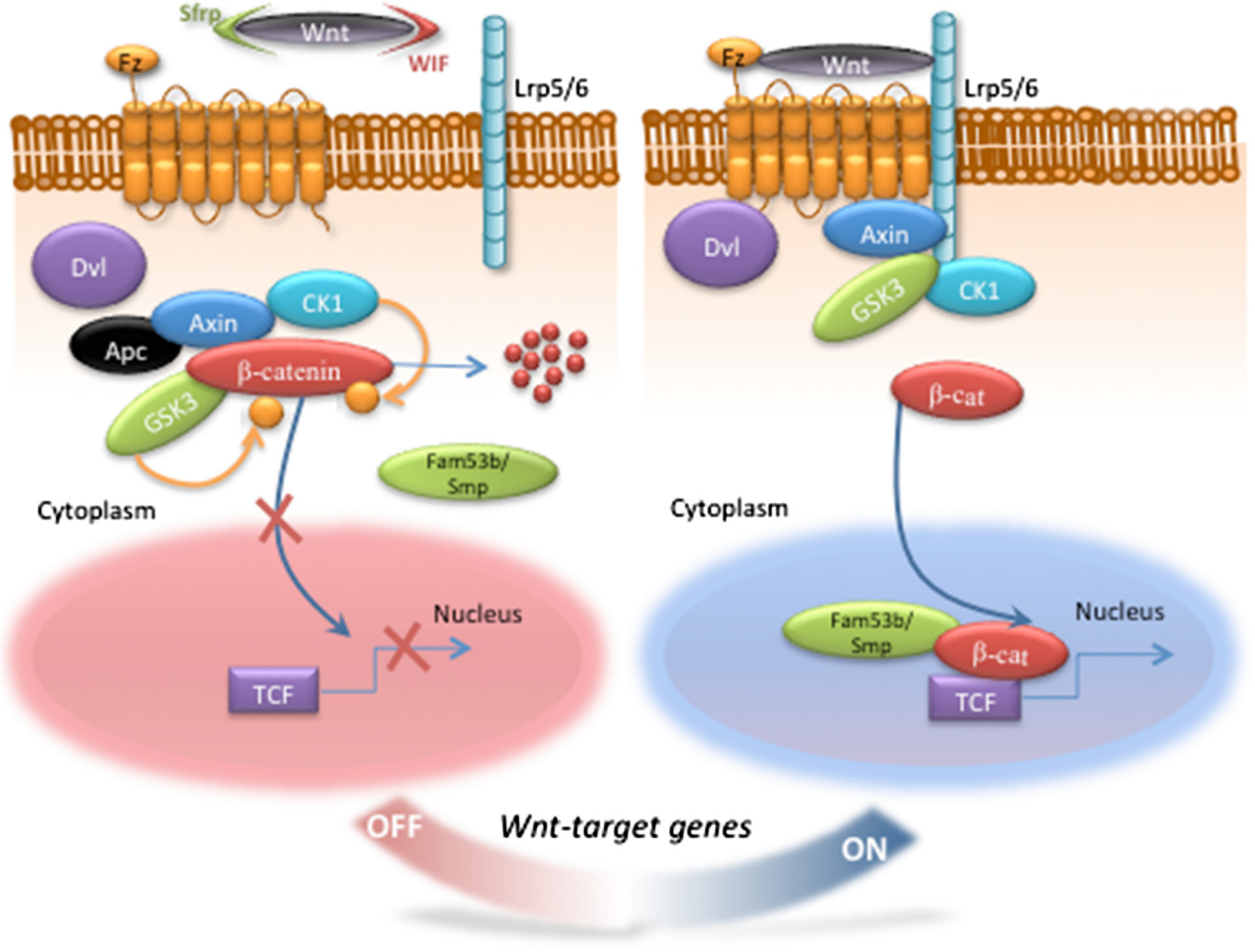
The Wnt/β-catenin signaling pathway. In the Wnt-off state, defined by the absence of an active Wnt ligand, β-catenin is phosphorylated by the destruction complex (formed from the two kinases Gsk3 and Ck1, the scaffolding protein Axin, and the tumor suppressor Apc) and degraded by the ubiquitin-proteasome pathway. In the Wnt-on state, active Wnt ligands interact with the Fz receptors and the Lrp5/6 coreceptor. Phosphorylation of Lrp5/6 by Gsk3 and Ck1 recruits Dvl and Axin to the receptor complex and hence inhibits the destruction complex. This, in turn, inhibits β-catenin phosphorylation and stabilizes β-catenin in the cytoplasm. β-catenin is then translocated into the nucleus, by a complex including Fam53b/Smp, and regulates target gene expression with the Tcf/Lef transcription factors. Many modulators including the inhibitors sFrps and Wif are known to tightly regulate the signaling cascade

Wnt/β-catenin signaling has vital functions during embryonic development, adult homeostasis, and tissue and organ regeneration [47, 48]. The pathway takes its name from a family of secreted glycoproteins, the Wnt proteins, which act as pathway ligands and from the downstream effector molecule, β-catenin [49, 50]. In the absence of an active Wnt ligand, *i.e.*, in the Wnt-off state, β-catenin is phosphorylated by a cytoplasmic complex of proteins (the “destruction complex”) that includes two serine/threonine kinases, namely Glycogen synthase kinase 3β (Gsk3β) and Casein kinase 1 (Ck1); the scaffolding protein Axin; and the tumor suppressor Adenomatous polyposis coli (Apc) (Fig. 1) [51, 52]. Phosphorylated β-catenin is ubiquitinated and targeted for degradation by the proteasome pathway [47, 50, 53, 54]. If active Wnt ligands are available, *i.e.*, in the Wnt-on state, they interact with Frizzled (Fz) receptors and the coreceptor Low-density lipoprotein receptor-related proteins 5/6 (Lrp5/6) [51, 55]. Lrp5/6 is then phosphorylated at its intracellular domain by Gsk3β and Ck1 in raft plasma membrane domains and internalized into intracellular vesicles [47, 56–58]. Lrp5/6 phosphorylation recruits the cytoplasmic scaffolding proteins Dishevelled (Dvl) and Axin to the receptor complex, leading to inhibition of the destruction complex and hence inhibition of β-catenin phosphorylation (Fig. 1) [51, 59]. This results in β-catenin stabilization in the cytoplasm and its translocation into the nucleus, which is in part mediated by Fam53b/Smp [60]. β-catenin regulates target gene expression with the transcription factors of the T cell factor (Tcf)/Lymphoid enhancer factor (Lef) family [47, 51, 61, 62]. The Wnt-off-state can also be brought about by a plethora of pathway inhibitors [63, 64], some of which act by binding to the Wnt ligands, such as secreted Frizzled-related proteins (sFrps) and Wnt inhibitory factor (Wif) [65].

## Wnt signaling in heart development

**Fig. 2:**
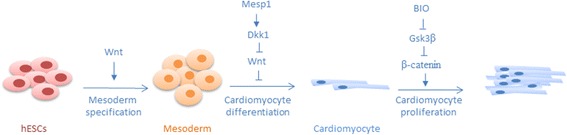
Roles of Wnt/β-catenin signaling during vertebrate heart development. Wnt/β-catenin signaling is required for heart development in a biphasic manner: while the activation of the pathway promotes mesoderm specification in early phases of hESC differentiation, it hampers CM differentiation at later stages. This late-stage suppression can act through the cardiac differentiation inducer Mesp1, which activates the Wnt inhibitor Dkk1. Wnt/β-catenin signaling can also regulate CM proliferation through Gsk3 by regulating β-catenin activity

Wnt/β-catenin signaling plays essential roles during vertebrate heart development [66]. Several studies have uncovered an inhibitory role of Wnt/β-catenin signaling in vertebrate heart specification, which is most dramatically revealed by the formation of ectopic hearts after conditional inactivation of β-catenin in the definitive endoderm of the mouse embryo [67]. However, during the last years, studies in zebrafish and mouse embryos and mouse and human embryonic stem cells (hESCs) have identified temporally distinct roles for Wnt/β-catenin signaling during vertebrate heart development. According to this model, the pathway induces cardiac specification during early developmental stages but inhibits it later [66, 68–72] (Fig. 2). Thus, treatment of early differentiating mouse or human ES cell cultures with Wnt ligands enhances CM formation via promotion of mesoderm specification, while later treatment with Wnts suppresses and the Wnt inhibitor Dickkopf1 (Dkk1) promotes CM differentiation [68, 70–72] (Fig. 2). Likewise, the cardiomyocyte progenitor cell (CMPC) marker Mesp1 drives cardiac differentiation of ES cells via directly activating Dkk1 expression [73]. These findings are highly relevant for efforts to produce human CMs for therapeutic transplantation from ES or induced pluripotent stem cells (iPSCs) in culture.

Less is known about potential roles of Wnt/β-catenin signaling in the regulation of CM proliferation. Treatment of neonatal or adult rat CMs in culture with the Gsk3 inhibitory small molecule BIO, which, among other effects, results in stabilization of β-catenin, can induce cell proliferation and mitosis [74]. Evidence that Wnt/β-catenin signaling regulates CM proliferation *in vivo* comes from a genetic study in mouse. Inactivation of the Hippo pathway, which restrains cell proliferation and thus controls organ size in *Drosophila*, in the embryonic mouse heart results in development of enlarged hearts which display increased numbers of CMs and increased CM proliferation [75]. Intriguingly, β-catenin levels are upregulated in such hearts and deletion of one copy of β-catenin can rescue the heart overgrowth phenotype. Thus, Wnt/β-catenin signaling might promote CM proliferation and is kept in check by the Hippo pathway. However, since Hippo and β-catenin were conditionally deleted in *nkx2.*5+ cardiomyocyte precursor cells early during heart development in this study, it remains possible that these pathways act on precursor cell proliferation or differentiation and do not directly control CM proliferation.

## Activation of the Wnt/β-catenin signaling pathway in response to cardiac injury

Heart injuries caused by myocardial infarction, pressure/volume overload, inflammatory diseases, cardiomyopathy, chronic hypertension, or congenital heart disease mostly affect the ventricles and lead to complex responses including scarring, fibrosis, and CM hypertrophy in mammals, which are generally referred to as ventricular remodeling. These changes cause a decline in the ventricular performance and contractile function, which can result in heart failure.

In addition to its role during cardiac development, Wnt/β-catenin signaling has also been implicated in regulation of cardiac remodeling and injury responses in mammals. While there is a body of work suggesting roles for Wnt/β-catenin signaling in protection of CMs from apoptosis and regulation of CM hypertrophy, there is little evidence supporting a direct role of the pathway in these processes. Furthermore, several of the Wnt pathway components shown to play a role in the cardiac injury response, in particular Gsk3 and sFrps, have additional functions unrelated to Wnt signaling, which perplex the situation.

Several studies have shown that the expression of Wnt ligands and of the feedback regulators Dkk1 and Dkk2 is induced in response to injury of the mouse heart [76–78]. Aisagbonhi and colleagues described induction of Wnt-2, Wnt-4, Wnt-10b, and Wnt-11 5 days post permanent left anterior descending (LAD) coronary artery occlusion in whole heart samples [76], while Duan and colleagues reported that Wnt-1 is robustly induced 2 days post injury and sustained in entire hearts in response to transient LAD occlusion, whereas Wnt-4 and Wnt-7a are only upregulated transiently at later stages [78]. It is surprising that these two studies only agree on Wnt-4 as an injury response gene. Further gene expression studies performed on transiently and permanently occluded hearts in parallel will be needed to clarify whether these discrepancies are due to the different injury models used. β-catenin levels have also been reported to increase in rat hearts starting 1 day post pressure overload induced by thoracic aortic constriction (TAC) [79]. Although functional roles of Wnt/β-catenin signaling during cardiac repair have been studied since about a decade, the sites of Wnt pathway activation in the injured heart have been analyzed only recently [76, 78, 80]. Using the Axin2-LacZ mouse, which expresses the marker LacZ under control of regulatory elements of Axin2, which is considered a universal Wnt feedback target gene, and thus serves as useful readout of β-catenin signaling activity, upregulation of pathway activity was reported after permanent LAD in endothelial cells, Sca-1 and c-kit-positive progenitors, fibroblasts, and smooth muscle cells; LacZ+ cells were mainly found in the infarct border zone starting at 7 days post injury (dpi), peaking at 14 dpi [80]. In addition, LacZ+ leukocytes started to accumulate at 3 dpi. Using the TopGAL mouse, upregulation of pathway activity could be detected 4 days after permanent LAD in subepicardial endothelial cells [76]. One week after injury, myofibroblasts expressing the TopGAL reporter accumulated in the infarct area, and genetic lineage tracing showed that these were derived from endothelial cells that appeared to undergo an endothelial-to-mesenchymal transition (EndMT) [76]. In contrast, TopGAL activity was reported in response to ischemia-reperfusion caused by transient LAD ligation primarily in the epicardium [76, 78, 80]. Since the different Wnt reporter mouse lines have been reported to display contrasting expression patterns in some tissues [81], a definitive determination of active sites of Wnt/β-catenin signaling in the injured mammalian heart will likely require the side-by-side comparison of different reporter mice using identical injury models and detection methods plus determination of endogenous target gene expression in isolated cardiac cell populations.

## A role for Wnt/β-catenin signaling in fibrosis

Although the studies by Aisagbonhi et al. and Duan et al. identified different primary sites of Wnt/β-catenin signaling induction in response to permanent LAD (subepicardial endothelium) and transient LAD (the epicardium), they surprisingly both define a functional role of Wnt/β-catenin signaling in promoting fibroblast formation from these cells in a process of endothelial or epithelial-to-mesenchymal transition (EndMT and EMT respectively) [76, 78]. In cultured endothelial cells, treatment with the Gsk3 inhibitor BIO, which induced direct β-catenin target genes and activity of the transcriptional Wnt reporter Topflash, was sufficient to induce EndMT and myofibroblast formation as evidenced by reduction of endothelial markers and induction of smooth muscle actin [76]. Likewise, Wnt-1 overexpression promoted EMT and fibroblast differentiation of cultured epicardial cells, while β-catenin deletion in epicardial cells reduced epicardial expansion and EMT in response to injury and resulted in reduced cardiac function [78]. Furthermore, β-catenin deletion in cardiac fibroblasts reduced their number in ischemia-reperfusion injured hearts and likewise caused cardiac dysfunction. Together, these data indicate that Wnt/β-catenin signaling promotes fibrosis in response to injury via inducing the transition to a mesenchymal state of both endothelial and epicardial cells; fibrosis, in turn, appears to be required to stabilize the wound and prevent cardiac dilation. Interestingly, Wnt/β-catenin signaling activation has also been reported in fibrotic diseases of the lung, liver, and kidney and Wnt/β-catenin signaling was found to be sufficient and essential for differentiation of fibroblasts into myofibroblasts and production of collagen by these cells [82, 83]. It will be very interesting to test whether Wnt/β-catenin signaling plays different roles in systems like the zebrafish or postnatal mice that activate a regenerative program rather than fibrosis in response to injury.

## A role for Wnt/β-catenin signaling in CM hypertrophy

Whether CMs activate Wnt/β-catenin signaling at later stages after infarction requires further investigation: while LacZ reactivity has been reported in heart regions containing CMs in the Axin2-LacZ mice, the identity of these cells was not confirmed by marker gene expression and the LacZ, which is targeted to the nucleus in these mice, was found in the cytoplasm in the presumptive CMs, raising questions about the validity of these results [80]. Furthermore, no CM expression was reported in the TopGAL mice [76, 78].

Thus, while activation of the β-catenin-dependent pathway in CMs in response to myocardial infarction *in vivo* has not been shown, functional evidence exists for an involvement of Wnt/β-catenin signaling in injury or stress-induced CM hypertrophy. However, conflicting data have been reported on whether β-catenin is required or actually inhibits CM hypertrophy. On one hand, several studies support a hypertrophy-promoting role for Wnt/β-catenin signaling. Conditional, CM-specific depletion of β-catenin in adult mice was found to impair CM hypertrophy in response to pressure overload induced by thoracic aortic constriction, while non-conditional transgenic overexpression of a dominant-negative Lef transcription factor in CMs throughout embryonic development resulted in CM hypotrophy [78]. Furthermore, transgenic overexpression of Gsk3 (which among other effects might inhibit β-catenin signaling) suppressed CM hypertrophy in response to stress [84]. β-catenin was found to be stabilized in cultured CMs in response to hypertrophic stimuli (phenylephrine or endothelin-1) due to inactivation of Gsk3 activity, but interestingly not in a Wnt pathway-dependent manner but rather via phosphorylation of Gsk3 at serine 9 by Protein kinase B (PtB) [85]. β-catenin knockdown also reduced phenylephrine-induced CM hypertrophy in cultured cells, possibly since upregulation of the fetal gene *anf* in response to phenylephrine is directly regulated by Lef1/β-catenin [86]. Contradicting a role for Wnt/β-catenin signaling in the promotion of hypertrophy are two studies using conditional deletion of β-catenin. β-catenin deletion in CMs did not impair CM hypertrophy in response to angiotensin II infusion [87]. On the contrary, mice expressing a constitutively active, stabilized β-catenin in CMs (achieved via conditional deletion of exon 3 of β-catenin, which codes for the domain phosphorylated by GSK3β) showed an abrogated hypertrophic response to angiotensin II [87]. Furthermore, the same mouse β-catenin deletion and overexpression models showed no alterations in CM hypertrophy at 2 and 4 weeks after infarct [88]. Currently, it remains unexplored whether the discrepancy between these and the above-mentioned studies showing a requirement for β-catenin for hypertrophy in response to thoracic aortic constriction can be explained by the different molecular responses induced by the different injury and stress models used.

Interestingly, treatment of cultured neonatal and adult mammalian CMs with the small Gsk3 inhibitor BIO, which results in β-catenin stabilization, has been found to be sufficient to induce CM proliferation [74]. Non-conditional Gsk3 knockout mice lacking Gsk3 throughout development display a CM hyperproliferation phenotype without defects in CM size [89]. Together with the data discussed above on the interaction of the Hippo and β-catenin pathways, these results indicate that Gsk3, possibly via reducing Wnt/β-catenin signaling, negatively regulates CM proliferation during embryonic development, a role that can be re-activated in adult CMs, at least in culture. This stands in contrast to the evidence supporting a positive role of Gsk3 inhibition and Wnt/β-catenin signaling in CM hypertrophy in the stressed adult myocardium. Additional conditional *in vivo* loss-of-function data will be required to clarify whether β-catenin function switches from CM proliferation-promoting in the embryo to hypertrophy-promoting in the adult.

## Wnt pathway-dependent and Wnt pathway-independent roles of sFrps

**Fig. 3:**
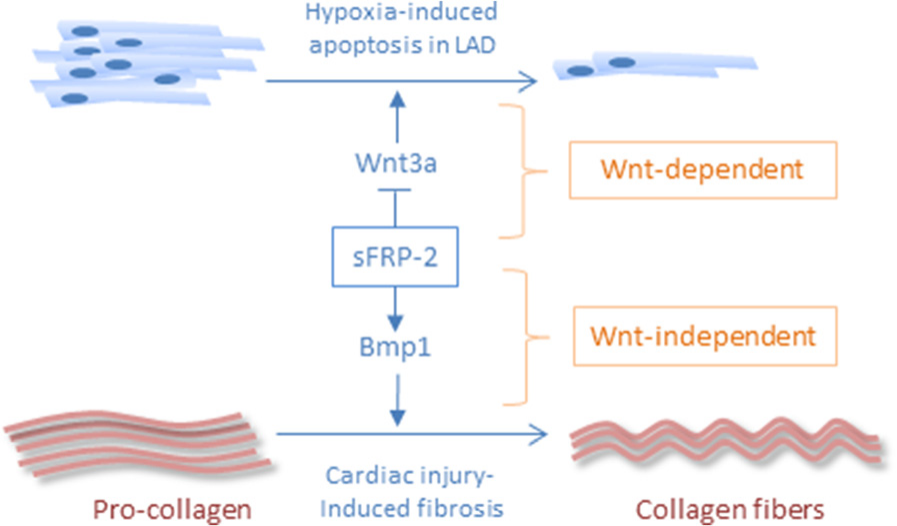
Wnt-dependent and Wnt-independent roles of secreted Frizzled-related proteins (sFrps). sFrp-2 can block hypoxia-induced CM apoptosis by activating a Wnt/β-catenin pathway. On the other hand, sFrp-2 can induce collagen deposition and fibrosis via enhancing Bmp1 function, which occurs through a Wnt-independent pathway

Expression of several sFrps, which can act as inhibitors of Wnt signaling pathways, is elevated in mouse models of myocardial infarction (MI) generated by LAD and in overload-induced failing human hearts [77, 90, 91]. However, functional data on the role of sFrps in mammalian heart remodeling and repair indicate that their role is highly complex and that they might in fact act as activators of Wnt/β-catenin signaling but also have important Wnt signaling-independent functions. Delivery of mesenchymal stem cells expressing Akt can improve cardiac function after infarction [92], and sFrp-2 secreted by these cells has been shown to be a key mediator of their positive effects [93]. Intriguingly, sFrp-2 was sufficient to protect CMs from hypoxia-induced apoptosis and surprisingly increased β-catenin levels in CMs. This result is in accordance with the proposal that sFrps, in addition to their role as Wnt ligand scavengers, are able to promote Wnt/β-catenin signaling by enhancing Wnt ligand diffusion [94]. Yet, how recombinant sFrp-2 might promote β-catenin accumulation in cultured CMs remains unexplained. Furthermore, Mirotsou et al. showed that hypoxic CMs upregulate Wnt3a expression, and Wnt3a treatment induced CM apoptosis, while sFrp-2 counteracted this effect [93], which surprisingly would indicate that Wnt3a inhibited and sFrp-2 activated Wnt/β-catenin signaling in this context (Fig. 3, Wnt-dependent route).

Constitutive overexpression of sFrp-1 in mice has been shown to reduce infarct size and cardiac function after coronary artery ligation or cryoinjury [77]. In contrast, sFrp-1 overexpression specifically in CMs did not affect infarct size or cardiac function in an ischemia-reperfusion (IR) model [95]. Rather, sFrp-1 expressed in CMs reversed the cardio-protective effect of pre-conditioning prior to IR and thus worsened infarcts after pre-conditioning, which stands in contrast to the positive effects observed after systemic sFrp-1 expression. Whether these differences are due to the different injury models used in these studies or reflect a cardio-protective function of sFrp-1 in non-CMs remains unexplained. sFrp-1 was proposed to activate Gsk3 function in the latter study using CM-specific sFrp-1 expression, but not via inhibition of Wnt/β-catenin signaling but by counteracting phosphorylation and thus inhibition of Gsk3 by a Pi3-kinase-protein kinase B pathway during cardiac pre-conditioning [95, 96]. This pathway regulates β-catenin-independent cardio-protective effects of Gsk3. Likewise, no effects on β-catenin levels were seen after CM-specific sFrp overexpression, indicating that sFrp-1 likely does not modify Wnt/β-catenin signaling in this context.

Clearly, additional experiments using transcriptional readouts of β-catenin activity in culture and *in vivo* plus loss-of-function experiments will be required to clarify whether Wnt/β-catenin signaling is cardio-protective and whether sFrps have an endogenous role as modifiers of this function. A recent report on the function of the secreted Wnt inhibitor Dkk3 actually showed that Dkk3 knockout mice had greater infarcts and aggravated left ventricular function after infarct, while Dkk3 overexpression protected from infarction [97]. This indicates that active Wnt/β-catenin would exacerbate cardiac injury and remodeling, while inhibition of β-catenin signaling by Dkk3 is cardio-protective.

Intriguingly, another Wnt signaling-independent function of sFrp has been uncovered in regulation of fibrosis in response to cardiac injury. Mammalian sFrp-2 has been found to enhance the function of the metalloproteinase Bmp1, which is a rate-limiting enzyme in the processing of pro-collagen during collagen fiber formation at physiological low concentrations (10–20 nM) [91] (Fig. 3, Wnt-independent route). Since collagen deposition is a hallmark of fibrosis occurring after cardiac injury, sFrp-2 might enhance fibrosis and thus worsen cardiac function after infarction. Indeed, fibrosis was found to be reduced and cardiac function to be improved in sFrp-2 knockout mice after coronary artery ligation [91]. Remarkably, at high concentrations, sFrp-2 was found to have the opposite effect: it inhibited Bmp1 activity and thus collagen deposition, and injection of recombinant sFrp-2 into the infarcted area of rats after coronary artery ligation reduced fibrosis and improved cardiac function [98]. Thus, sFrps can act through Wnt-dependent or Wnt-independent pathways and appear to positively or negatively regulate the cardiac response to different injury types in a context-dependent manner (Fig. 3).

## A role for Wnt/β-catenin signaling in response of cardiac progenitor cells to injury

As outlined above, conflicting data exist on the *in vivo* importance of cardiac progenitor cells for CM formation during adult homeostasis and in response to heart injury [22, 24]. Some evidence exists for a role of Wnt/β-catenin signaling in regulation of progenitor cell differentiation. Zelarayan et al. reported that conditional depletion of β-catenin from the adult mouse myocardium using a MHC-Cre driver line resulted in improved survival and left ventricular function 4 weeks after chronic LAD [88]. The Cre driver was found to be active in a population of progenitor cells (as defined by absence of expression of cardiac Troponin T (cTnT) but presence of proliferation markers and the embryonic CM transcription factors Gata4 and Tbx5). β-catenin depleted infarcted hearts showed increased numbers of cTnT-positive CMs in subepicardial and subendocardial positions, which the authors proposed not to be due to increased survival, proliferation, or hypertrophy of existing differentiated CMs. Rather, they found that isolated progenitor cells from β-catenin depleted hearts showed increased tendency to differentiate into cTNT+ cells in coculture with CMs [88]. Thus, β-catenin might inhibit differentiation of cardiac progenitor cells in the injured heart, consistent with the negative role of Wnt/β-catenin signaling in the later phases of heart development. Another recent study found that injection of recombinant Wnt3a protein into infarcted mouse hearts resulted in an increase in infarct size and worsened cardiac performance, and it was suggested that this is due to a negative influence of Wnt3a on proliferation of cardiac side population (sca1+, c-kit−, isl−) progenitor cells [99]. The *in vivo* relevance of this finding however has not been assessed using loss-of-function experiments.

## Therapeutic interventions on progenitor cells based on modulation of Wnt/β-catenin signaling

Human pluripotent stem cells (hPSCs), including hESCs and iPSCs, provide a potentially unlimited supply of progenitor cells that can differentiate into the vast majority of somatic cell types including CMs [100–102]. Temporal modulation of Wnt/β-catenin signaling via small molecule inhibitors or shRNA knockdown has been shown to affect the differentiation of hPSCs to CMs. Lian and colleagues found that short hairpin RNA (shRNA) knockdown of β-catenin during the early hPSC differentiation period arrested CM specification while small molecule mediated inhibition of GSK3 within the same period promoted it in multiple hPSC lines [103]. Inhibition of Wnt/β-catenin signaling during later phases of cardiac differentiation, however, can promote cardiac differentiation in hPSCs and generate functional CMs [104–106] as it does during development [66, 68–72]. Wnt/β-catenin signaling has also been reported to contribute to CM differentiation in progenitor cells, other than hPSCs. Cardiogenol C, a small molecule that was shown to induce ES cells to differentiate into CMs [107], was sufficient to promote hair bulge progenitor cells (HBPCs) to transdifferentiate into CM-like cells most likely by activating the Wnt/β-catenin pathway through suppression of Kremen1 [108]. Early treatment of human iPSCs with Bmp-4 followed by late treatment with small molecule Wnt inhibitors caused a remarkable increase in the total yield of biologically functional CMs, suggesting that modification of Bmp-4 and Wnt/β-catenin signaling pathways in human iPSCs might create an efficient platform to produce new CMs after cardiac injuries [109]. Similarly, the Cardionogen family of chemicals, which inhibit β-catenin-dependent transcription in murine ES cells and zebrafish embryos, modulated cardiogenesis in a biphasic manner and, while failing to induce CM differentiation during the initial ES cell differentiation period, increased the number of CMs by expanding the cells in both systems [110]. Moreover, another Wnt/β-catenin pathway inhibitor molecule, pyrvinium, was found to reduce adverse cardiac remodeling in mice infarcted by LAD after intracardiac injection, increase proliferation of differentiated CMs, and hence promote wound repair [111]. While modulation of the Wnt/β-catenin pathway thus appears to promote the cardiac differentiation of hESCs and iPSCs and tissue repair following heart injury, it should be noted that the molecular mechanisms of these effects remain unclear.

## Heart regeneration in the zebrafish model

In contrast to the limited regenerative ability of the adult mammalian heart, urodele amphibia and teleost fish retain regenerative capacity of the heart throughout adult life [3, 112]. Most of our knowledge about the mechanisms underlying heart regeneration comes from studies using zebrafish. While teleost fish have only a two-chambered heart (one ventricle, one atrium), the cellular composition, physiology, and development of the fish heart are well conserved with that of mammals. Due to a wealth of accumulated knowledge about heart development and the availability of many molecular, genetic, and genomic tools, zebrafish is an excellent model to study the cellular and molecular mechanisms of heart regeneration [113–115]. Furthermore, due to their short generation time, small size, and cheap husbandry, a high number of individual fish can be studied and transgenic and mutant lines can be produced quickly and at relatively low cost. The adult zebrafish heart is also easily accessible for surgical or other experimental manipulations, and the animals are highly tolerant to experimental cardiac injury [2].

Adult zebrafish hearts have been shown to display remarkable regenerative capacity in response to three types of injury: surgical removal of up to 20 % of the apex of the ventricle, cryoinjury-induced necrosis of up to 25 % of the apical ventricular region [9, 11, 116], and even mosaic ablation of up to 70 % of CMs caused by transgenic expression of diphtheria toxin [12]. In all three injury regimes, lost CMs are being replaced within 30 to 120 days and no or little permanent collagen-rich scar tissue forms.

While assays for heart function, in particular assessment of cardiac hemodynamics using ultrasound, are difficult due to the small size of the zebrafish heart and thus not trivial to perform [117–119], there is increasing evidence for functional recovery during zebrafish heart regeneration. Myocardium at the apex of the ventricle, which presumably has formed anew during regeneration in response to ventricular resection, was found to be electrically coupled to the rest of the ventricle, indicating functional recovery [14]. Likewise, coupling measured by optical mapping was found to be restored to pre-injury levels 45 days post genetic ablation of CMs, as was a lengthening in action potential duration occurring after ablation as measured at the tissue level [12]. Electrocardiogram recordings indicate that prolonged QT intervals were measured during recovery after cryoinjury [9]. However, another report found that regenerated hearts retained a prolonged QT interval after ventricular resection [120]. Side-by-side comparison of different injury models in one study as well as measurements of the electrophysiological properties of individual CMs [121] will be instrumental in clarifying whether zebrafish can completely recover the electrophysiology of the heart. Measurements of cardiac performance based on echocardiography indicate that heart function does recover; yet this might take longer than morphological regeneration. Here, one study reported that heart function after cryoinjury recovered only within 180 days [122], while another study showed that pumping fraction based on relative fractional volume shortening recovered within 60 days in cryoinjured hearts, while ventricular wall motion remained altered even after 140 days [123]. Overall, these data indicate that zebrafish can not only regenerate heart tissue architecture but also function, albeit it remains somewhat unclear how complete the functional regeneration is.

The cellular mechanisms activated after wounding and underlying heart regeneration appear to be highly similar between the injury models. A few days after injury, transcriptional responses occur in the entire epicardium, including upregulation of genes normally expressed during development (including Wilms’ tumor 1b), which is followed by proliferation and expansion of the epicardium into a multilayered structure [9, 11, 12, 116, 124]. Interestingly, similar epicardial responses occur in regenerating neonatal mouse hearts [13]. Genetic ablation of the epicardium has shown that it is essential for myocardial regeneration, but the nature of the molecular signals mediating its effects on CMs during zebrafish heart regeneration has not been elucidated [125]. In infarcted mouse hearts, epicardial Wilms’ tumor 1 (*wt1*) expression can also be re-activated by experimental treatment with the peptide thymosin 4 and *wt1*-positive cells have been reported to represent multipotent progenitors that can give rise to CMs after injury, albeit at a low frequency [126]. In contrast, in the regenerating zebrafish heart, genetic lineage tracing of epicardial cells identified by expression of *tcf21* showed that epicardial cells give rise to perivascular cells but not CMs [127]. Thus, naturally occurring myocardial regeneration in the zebrafish occurs without a cellular contribution of the epicardium. While regenerating CMs have initially been proposed to be derived from progenitor cells that do not have CM character [128], genetic lineage tracing data using the Cre-Lox system has rather indicated that the entire regenerated myocardium is derived from existing, differentiated CMs [14, 15]. In particular, a subepicardial population of CMs characterized by expression of a transgene driven by a *gata4* promoter fragment appears to produce the bulk of the regenerated myocardial tissue [14, 129]. Thus, CMs dedifferentiate in response to injury, re-enter the cell cycle, and proliferate to replace the missing tissue.

Although a large body of data shows that Wnt/β-catenin signaling plays important roles in mammalian cardiac remodeling and regeneration processes occurring in response to heart injury, its potential role in regulation of zebrafish heart regeneration remains to be tested. We have previously found that a transgenic reporter of Wnt/β-catenin signaling is upregulated in the injured ventricle after ventricular resection [130]. Besides, a novel Wnt target gene *simplet* (*smp*), which we have identified to be necessary for β-catenin-dependent signal transduction [60], is also transcriptionally activated in the zebrafish heart after amputation injury [131]. Thus, while Wnt/β-catenin signaling appears to be linked to heart regeneration in zebrafish, our efforts to test its function in heart regeneration using transgenic lines allowing for heat shock-inducible overexpression of Wnt modifiers have been confounded by the realization that heart regeneration can be impaired by the stress inflicted by heat shocking the fish (data not shown). Thus, these tools, which we have successfully used to uncover an essential function for Wnt/β-catenin signaling in the regulation of zebrafish fin regeneration [130, 132], are not ideal for the analysis of heart regeneration. Likely, studies into the role of Wnt/β-catenin signaling in zebrafish heart regeneration will benefit from further technological advances allowing for alternative methods of inducible manipulation of gene expression [133, 134].

## Conclusion

Heart regeneration is a complex process that only occurs in lower vertebrates, but not in mammals. Several processes such as dedifferentiation, proliferation, redifferentiation, and patterning should take place in concert in a highly regulated manner. Wnt/β-catenin signaling is crucial for orchestration of the regeneration process in other systems [132, 135]. Therefore, further studies will likely also uncover a role for Wnt/β-catenin signaling in lower vertebrate heart repair. Whether its role will be similar or different to the rather complex functions that Wnt/β-catenin signaling plays after mammalian heart injury will be interesting to study. Regenerating organisms such as zebrafish provide an excellent tool to address these issues, and regenerative therapeutic applications will surely benefit from a more widespread use of this model.
